# Visibility from Roads Predict the Distribution of Invasive Fishes in Agricultural Ponds

**DOI:** 10.1371/journal.pone.0099709

**Published:** 2014-06-12

**Authors:** Toshikazu Kizuka, Munemitsu Akasaka, Taku Kadoya, Noriko Takamura

**Affiliations:** 1 Center for Environmental Biology and Ecosystem Studies, National Institute for Environmental Studies, Tsukuba, Ibaraki, Japan; 2 Faculty of Agriculture, Tokyo University of Agriculture and Technology, Fuchu, Tokyo, Japan; University of Western Ontario, Canada

## Abstract

Propagule pressure and habitat characteristics are important factors used to predict the distribution of invasive alien species. For species exhibiting strong propagule pressure because of human-mediated introduction of species, indicators of introduction potential must represent the behavioral characteristics of humans. This study examined 64 agricultural ponds to assess the visibility of ponds from surrounding roads and its value as a surrogate of propagule pressure to explain the presence and absence of two invasive fish species. A three-dimensional viewshed analysis using a geographic information system quantified the visual exposure of respective ponds to humans. Binary classification trees were developed as a function of their visibility from roads, as well as five environmental factors: river density, connectivity with upstream dam reservoirs, pond area, chlorophyll *a* concentration, and pond drainage. Traditional indicators of human-mediated introduction (road density and proportion of urban land-use area) were alternatively included for comparison instead of visual exposure. The presence of Bluegill (*Lepomis macrochirus*) was predicted by the ponds' higher visibility from roads and pond connection with upstream dam reservoirs. Results suggest that fish stocking into ponds and their dispersal from upstream sources facilitated species establishment. Largemouth bass (*Micropterus salmoides*) distribution was constrained by chlorophyll *a* concentration, suggesting their lower adaptability to various environments than that of Bluegill. Based on misclassifications from classification trees for Bluegill, pond visual exposure to roads showed greater predictive capability than traditional indicators of human-mediated introduction. Pond visibility is an effective predictor of invasive species distribution. Its wider use might improve management and mitigate further invasion. The visual exposure of recipient ecosystems to humans is important for many invasive species that spread with frequent instances of human-mediated introduction.

## Introduction

Invasive alien species are widely acknowledged as a major threat to the biodiversity of native species. Controlling species invasion is a priority for conservation of native assemblages [1], [2]. Predicting which sites will be susceptible to the introduction and establishment of invasive species and which factors will be associated with their establishment can aid in controlling species invasion [3], [4]. When invasive species have high rates of propagule supply, propagule pressure can play a much more prominent role in predicting their distribution than habitat characteristics do [5].

Propagule pressure of an invasive species is determined by the extent of natural dispersal and human-mediated introduction. When physical barriers limit the extent of natural dispersal, human-mediated movement of a species can strongly affect the propagule supply. The distribution of aquatic organisms is often limited by the extent and availability of natural dispersal. Therefore, human-mediated movement of species is an important structuring influence.

Because of the difficulties in estimating the actual locations, size, and number of instances of human-mediated introduction into recipient areas, various surrogate indicators such as human population [6], [7], roadways [8]–[10], and urban land use [11]–[13] have been used to analyze the distributional patterns of invasive plant and animal species. For example, McKinney [14] and Gido et al. [5] found greater numbers of invasive species that had some value to humans (e.g., bait-bucket and sport fish) in highly populated areas at national and state scales in the United States. These studies demonstrated the effectiveness of surrogate indicators of propagule pressure. Their results indicated that the potential accessibility to recipient areas can be a key factor for human-mediated introduction. However, on local scales, for which traditional indicators such as human population, road density, and proportion of urban land-use area often do not show large spatial variation, these indicators might not reflect potential accessibility effectively. In such cases, accessibility probably depends on additional local conditions such as road-use types, travel time and cost, fences, and the visibility of recipient areas. Among them, visibility, the visual exposure of an area to humans, is expected to influence the probability of propagule supply, particularly for species that are introduced intentionally by humans after searching for a recipient area (e.g. a water body for game fishes) in the field. Even if the recipient area is surrounded by high-density roads, accessibility is assumed to be lower in cases where visual exposure to roads is obstructed by rough terrain or blocks of buildings. In such cases, visibility is likely to be more appropriate for evaluating accessibility than road density is, but no examination of their effectiveness has ever been reported in the literature.

Here, we used the visual exposure of a recipient area to roads, its visibility, as a surrogate of propagule pressure to test the effectiveness of visibility for predicting the distribution of invasive species. We also compared this visibility variable with other traditional indicators such as road density and the proportion of urban land-use area (defined as the urban ratio). Visibility has been used widely in archaeological studies [15] and urban landscape planning [16]. The emergence of powerful analytical tools in geographic information system [GIS] software, coupled with computer graphics techniques and increasingly large-scale and higher-resolution terrain data has facilitated the quantification of visibility and has led to its application in various areas of research [17]. Recently, visibility has been applied for ecological studies in relation to habitat selection of wildlife [18], but its application to propagule pressure of invasive species has not been reported.

We used agricultural ponds and two predatory invasive fish species, Bluegill sunfish (*Lepomis macrochirus*) and Largemouth bass (*Micropterus salmoides*), to test the effectiveness of visual exposure to roads. Unlike open terrestrial ecosystems, agricultural ponds are more closed systems; the respective ponds have limited connectivity. For that reason, such systems are appropriate for testing the contribution of human-mediated introduction to propagule pressure. Agricultural ponds and small reservoirs support diverse populations of aquatic animals and plant species [19]–[22]. They are the most important habitats in terms of both local and regional biodiversity among several aquatic habitat types (i.e., lakes, ponds, ditches, streams, and rivers) in agricultural landscapes [23]. In such shallow water ecosystems, predatory invasive fish species negatively affect macroinvertebrate fauna and indigenous fish species through direct predation and competition [24]. Predicting the distribution of invasive fish species is an important task for biodiversity conservation of pond ecosystems.

## Materials and Methods

### Study area

**Figure 1:**
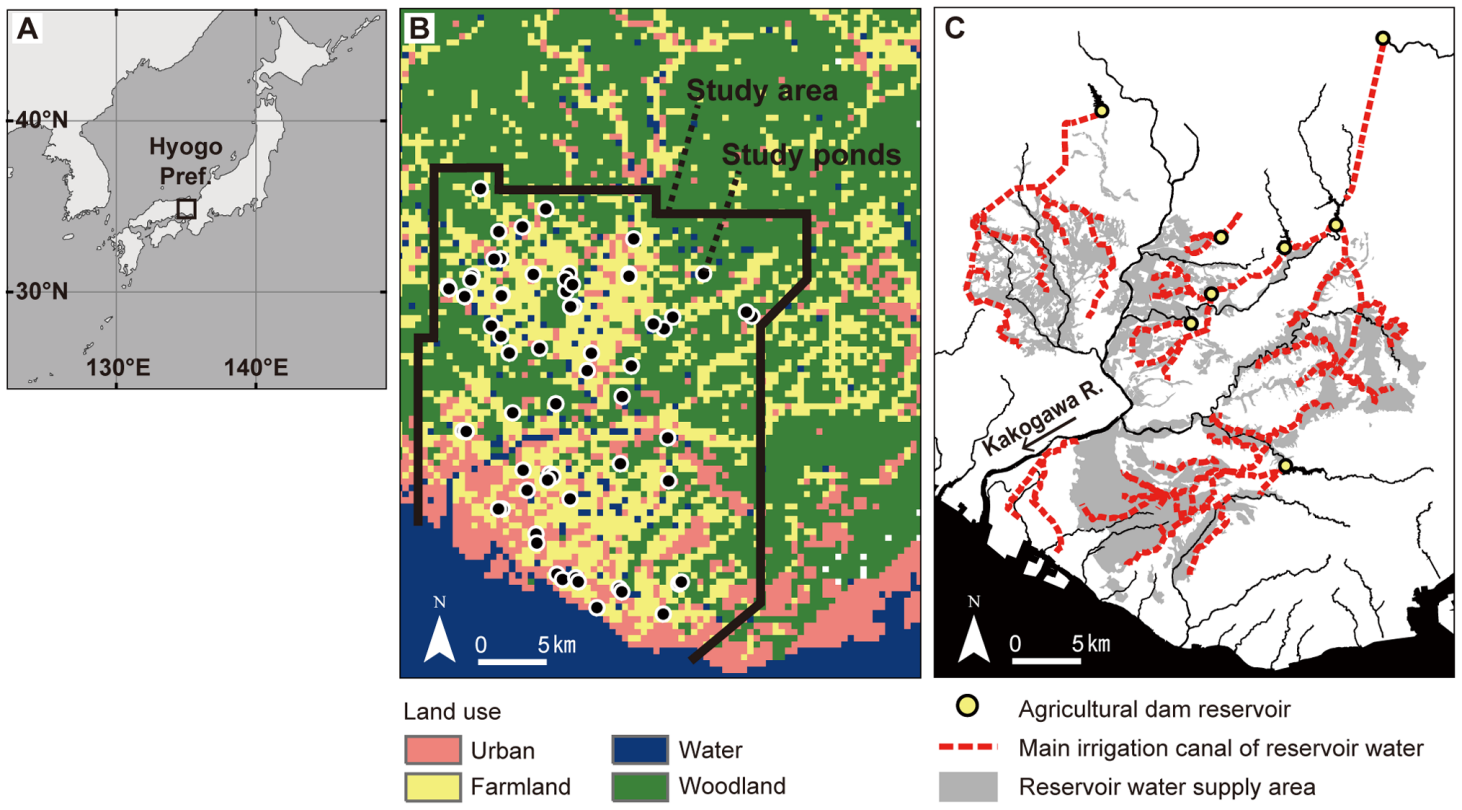
Description of study area. A) Locations of Hyogo Prefecture, Japan, B) study area and ponds with land use, C) agricultural dam reservoirs, irrigation canals, and reservoirs' water supply area. Land use in (B) and irrigation canals in (C) are described respectively using Land utilization segmented mesh data with 100 m meshes (created in 2009; National Land Information Division, National and Regional Policy Bureau, Ministry of Land, Infrastructure, Transport and Tourism, Tokyo, Japan) and the 1/25,000 irrigation canal network maps (provided by Japanese Institute of Irrigation and Drainage, Tokyo, Japan).

This study examined an area of approximately 1000 km^2^ in southwestern Hyogo Prefecture, Japan (Fig. 1). Many agricultural ponds have been created in Hyogo Prefecture (8,395 km^2^) to irrigate paddy fields. More than 55,000 agricultural ponds, corresponding to about 20% of all ponds in Japan, were recorded in the 1950s [25]. Nevertheless, more than 11,000 of those ponds had been lost by 1997, mainly as a result of urban or residential development [26]. Even where ponds have not been destroyed, their biodiversity has decreased drastically during recent decades [27].

The study area, with elevations of 0–640 m a.s.l., includes mountainous (9% of the study area), hilly (31%), plateau (29%), and lowland (31%) areas (1/200,000 landform classification map provided by the Japanese Ministry of Land, Infrastructure, Transport and Tourism [MLIT], Tokyo, Japan). The study area has a warm temperate climate with an annual mean temperature of 14.4 °C (minimum, 3.5 °C in January; maximum, 26.4 °C in August) and mean annual precipitation of 1198.3 mm (data provided by the Miki Climatological Observatory located within the study area at 145 m a.s.l.). The predominant land uses are woodland (36%), farmland (34%), and urban land (18%) (Fig. 1B; Land utilization segmented mesh data with 100 m meshes; created in 2009; National Land Information Division, MLIT, Tokyo, Japan). The southern coastal region has been particularly urbanized. The study area, which has an average population density of 1,206 people per square kilometer, is adjacent to the second largest metropolitan area in Japan (Osaka) on the east.

Generally, agricultural ponds are constructed by banking up streams or spring water. For that reason, each pond has an original catchment upstream. The pond water resource depends fundamentally on rainwater that the catchment receives. After the late 1940s, however, massive water resource development projects including dams and canals changed irrigation systems and diversified the ponds' water resources. Now, reservoir and river water are supplied to ponds as necessary via canal networks with main canals of approximately 3 m width and 2 m depth [28]. In the study area, eight agricultural dam reservoirs were constructed during 1940–2000 [29] (Fig. 1C). Some ponds receive reservoir water indirectly from upstream ponds and farmlands in their catchments that are connected directly to the reservoirs' water canals.

### Invasive fish species

Bluegill and Largemouth bass, freshwater fish native to North America, are listed among the IUCN 100 worst invasive species in the world. Each has a long and successful invasion history in Japan's lentic environments. Each species is listed as an Invasive Alien Species [IAS] under the Japanese IAS Act adopted in 2005; it is strongly prohibited to transport or stock these living organisms without permission from competent authorities. Negative effects of these invasive fish on lake and pond ecosystems in Japan have been reported from many studies. The abundance of Bluegill and Largemouth bass has negatively affected total richness, endangered species, and functional diversity of various taxa including native fishes, aquatic macrophytes, Odonata, and benthic macroinvertebrates in agricultural ponds [24], [30]. Experimental approaches have revealed direct predation and competition in addition to cascading effects through the chain of bass–crayfish–macrophyte [31]. Bluegill juveniles feed on crustacean zooplankton, which has caused an increase of phytoplankton biomass as a result of reduced grazing pressure [32]. Through alterations of species composition, trophic structure, and ecosystem features, these invasive fishes adversely affect fishery production in many lakes [33].

Bluegill were first introduced to the Freshwater Fish Research Institute of the Fisheries Agency of Japan in 1960 [34]. Some of these offspring were released into the wild, although others were provided to prefectural experimental stations and fishermen for aquaculture as a potential new food resource [35]. Although aquaculture never became established as an industry, their rapid spread became apparent across the country after the 1980s, which was likely related to their stocking by anglers coupled with Largemouth bass as forage. Largemouth bass were first introduced into Japan in 1925 and again in 1972 as a food and game species [36], [37]. Their distribution spread rapidly to other water bodies including agricultural ponds throughout the Japanese Islands after the late 1960s as sport fishing became popular.

Reportedly, Bluegill and Largemouth bass were first introduced, respectively, into the wild in Hyogo Prefecture by 1987 [38] and by 1964 [39]. The population of anglers seemed to decrease after the peak in the late 1990s in the area. Still now, however, agricultural ponds are often used for sport fishing throughout the prefecture. Consequently, human-mediated introduction for sport fishing is assumed to be one factor influencing the present distributions of these invasive species. Anglers generally access agricultural ponds using private vehicles. Therefore, visual detection of the ponds from surrounding roads is regarded as important for access to the ponds. Aside from human-mediated introduction, natural dispersal from reservoirs established upstream and from other ponds via canals occurs. Younger fish, which have less swimming capability, can readily disperse in that manner [40]. Connections with upstream dam reservoirs where the invasive fish reproduce are regarded as another important factor affecting their distribution in agricultural ponds [26]. Additionally, the natural spread of invasive fish might occur via surrounding rivers that connect ponds as water corridors [41].

Largemouth bass are piscivores that can eat fish, insects, crayfish, and zooplankton in agricultural ponds [42]. In contrast, Bluegill are omnivores known to have trophic polymorphism because of their different requirements for efficient resource utilization [43]. In addition to their wide feeding niche, Bluegill show high physiological and behavioral adaptability to changing environments [44], [45].

### Selection of study ponds and invasive species survey

The visibility of ponds from roads is affected by landforms and land use [46]. Therefore, we chose 64 ponds based on combinations of landform classifications (lowland, 17 ponds; hilly, 12 ponds; plateau, 35 ponds) and the predominant land use (woodland, 22 ponds; farmland, 31 ponds; urban, 11 ponds) in areas surrounding the ponds. The selected ponds had surface areas of 685 m^2^ to 111,626 m^2^ (mean±SD: 11,052±14,563 m^2^), with maximum depths of 0.3–6.0 m (2.2±1.3 m). The average pond elevation was 57±33 m a.s.l. Based on interviews with pond managers, we confirmed that the managers and farmers had never stocked their own ponds with largemouth bass or bluegill.

To catch invasive fishes, a Y-shaped fixed net (6.0-m sleeve and 0.7-m open mouth; 4-mm mesh) was set for one night in the littoral zone of each pond during 19 September – 5 October 2006 or 4–11 October 2007. For the same time period, five box-net traps (0.4 m × 0.25 m × 0.4 m with 2-mm mesh) were set for one night along a transect extending from the shallower littoral zone to the deeper limnetic zone in each pond. Furthermore, invasive fishes were sampled using standard D-frame nets (0.35 m open mouth with 2-mm mesh) mainly in littoral zones with aquatic plants (3–12 points of each pond). We counted all individuals of invasive fish species caught in the nets and traps.

We obtained permits for the survey from each pond manager in conjunction with the Agricultural and Environmental Affairs Department, Hyogo Prefecture Government. Surveyed ponds did not involve protected areas and species that required permits for sampling. The sampled invasive alien species were processed in accordance with the Japanese IAS Act.

### Environmental characteristics

We assessed eight candidate characteristics that putatively affect the distributions of the invasive fish species. (1) Visibilities of ponds from surrounding roads, (2) connectivity with upstream dam reservoirs, and (3) river densities surrounding ponds were examined as landscape characteristics related to propagule supplies from outside the ponds. For comparison with visibility as a surrogate indicator of human-mediated introduction, (4) the road densities and (5) urban ratio of areas surrounding ponds were assessed. In addition, (6) the surface area, (7) chlorophyll *a* concentrations (chl.*a*), and (8) the presence or absence of pond water drainage and drawdown during winter were examined as site characteristics related to the establishment of the invasive fish species. We used surface area as a site characteristic because ponds with greater surface area are assumed to have various microhabitats from shallower littoral zones with aquatic plants to deeper limnetic zones, which provide better opportunities for the establishment of large fishes by supporting spawning and feeding [47]. Furthermore, we used chl.*a* as an indicator of eutrophication. It affected the populations and communities of fish through changes in energy availability, turbidity, and dissolved oxygen concentrations in water [48], [49].

Visibility is an indicator of how much of a target pond can be seen from surrounding roads. Visibility was assessed objectively by generating viewshed maps using ArcGIS 10.0 with the extension 3D Analyst (ESRI, Redlands, USA). They cover the area that is visible from observation points in a three-dimensional digital geographical surface, as represented by Digital Surface Models [DSMs]. We set observation points at 100 m intervals along road polylines derived from the 1/2,500 Digital Map (Spatial Data Framework; published in 2006, Geospatial Information Authority [GSI], Tsukuba, Japan). These observation points were positioned 1.5 m above the surface. Sight lines were drawn with the longest distance of 500 m from each observation point. No constraint was set on the height or direction of sight lines. We used DSMs with 1-m spatial resolution derived using photogrammetry software (SOCET SET v5.5; BAE Systems plc, London, UK) from 1/15,000 aerial photographs taken in 2007. We calculated the number of observation points from which each 1 m^2^ parcel was visible (defined as viewshed points) and evaluated the visibility of each pond by aggregating the viewshed points of all parcels located within the polygon of a pond's surface area including the vegetated littoral zone. These pond polygons were delineated based on the orthorectified images of the aerial photographs described above and based on the latest 1/2,500 topographic maps provided by local governments.

We calculated the road densities and urban ratios for buffer areas within 500 m from the edges of pond polygons, which was the same extent as the longest distance of sight lines for visibility analysis. Total lengths of road polylines and total areas of urban land use, for which both data sources were described above, were divided by the buffer area of each pond.

Connectivity between study ponds and upstream dam reservoirs was assessed based on the 1/100,000 reservoir water supply area maps, the 1/25,000 irrigation canal network maps (provided by Japanese Institute of Irrigation and Drainage, Tokyo, Japan), and a list of irrigation facilities. We treated connectivity as a categorical variable in the following statistical analysis: 0, ponds in which reservoir water did not flow; 1, ponds in which reservoir water flows indirectly via upstream farmlands or ponds; 2, ponds in which reservoir water was supplied directly from irrigation canals.

We used 1/25,000 National Land Numerical Information Rivers Data (published in 2012; National Land Information Division, MLIT, Tokyo, Japan) to calculate the river densities surrounding ponds. Graded buffers (5, 10, 25, 50, 100, 250, 500, and 1000 m from the edges of pond polygons) were delineated. Then the total lengths of river lines were divided by each buffer area.

Surface areas of ponds were calculated based on the pond polygons described above. We measured chl.*a* of ponds' water in the summer (end of July in 2006 or 2007) at the approximate center of each pond using a standard method (details in Kadoya et al. [24]). Some pond managers drain pond water to check and repair levees, to improve water quality, and to eradicate invasive fish during winter (November–March), when irrigation water is not required for rice cultivation. Based on interviews with pond managers, we examined whether water was drained periodically or not at each pond, and treated that information as a binary variable in subsequent analyses.

### Statistical analysis

We used a binary classification tree [50], [51] to analyze the presence and absence of invasive fish species in the ponds as a function of six environmental factors: visibility, river density, connectivity with upstream reservoirs, pond area, chl.*a*, and pond drainage. To test the importance of visibility as a surrogate indicator of human-mediated introduction, each of the three indicators (visibility, road density, and urban ratio) was alternatively included as environmental factors of classification tree. Then we compared model performance among these trees for each invasive fish. Splitting variables and the split criteria were determined statistically in each tree. Ten-fold cross-validation was used to obtain estimates of cross-validated relative errors of these trees [52]. These estimates were then shown against tree size. Then the optimal tree was chosen based on the 1–SE rule, which minimizes cross-validated error within one standard error of the minimum [50]. To eliminate the influence of random split of samples in the cross-validation, a series of 50 cross-validations was run for each invasive fish. Then the modal (most likely) single optimal tree was chosen for description. We calculated the overall misclassification, sensitivity (the ability of the model to predict that an invasive fish is present when it is) and specificity (the ability of the model to predict that an invasive fish is not present when it is not) of the optimal tree for all datasets. We used the R statistical environment (R ver. 2.15.0) [53] and the rpart package (rpart ver. 3.1–53) [54] to build models and to evaluate their performance.

## Results

### Invasive fish species

Bluegill and Largemouth bass were caught respectively in 40 ponds (63% of all studied ponds) and in 12 ponds (19%) (Table 1). Of all ponds, 67% (43 ponds) supported at least one of the invasive fish species. Among them, almost all ponds supported only Bluegill (31 ponds). Three ponds supported only Largemouth bass. Nine ponds supported both species.

**Table 1:** Number of ponds in which Bluegill (*Lepomis macrochirus*) or Largemouth bass (*Micropterus salmoides*) were caught, their percentages for total studied ponds, and summaries of total individuals caught in fixed nets, box-net traps, and D-frame nets in 64 agricultural ponds in Hyogo Prefecture, Japan.

	Species presence		Individuals			
Invasive alien fish species	Number of ponds	%	Min.	Max.	Mean	Total
Bluegill (*Lepomis macrochirus*)	40	63	0	246	33.6	2149
Largemouth bass (*Micropterus salmoides*)	12	19	0	407	6.7	428

### Environmental characteristics

**Figure 2:**
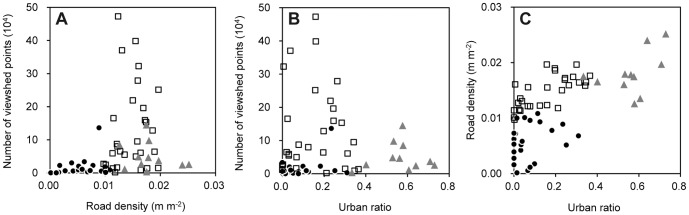
Relation between number of viewshed points, road density and urban ratio. Road density and urban ratio represent the total length of road line for 500(m m^−2^) and the proportion of urban area for a 500 m buffer area, respectively, for 64 agricultural ponds in Hyogo Prefecture, Japan. Closed circles, open squares, and grayed triangles represent dominant land use types for 500 m buffer areas corresponding respectively to woodland, paddy field, and urban areas.

The visual exposure of ponds to roads (viewshed points), road density, and the urban ratio varied greatly among ponds, ranging 0–472,752 (median 26,239), 0.14–25.18 (12.21; unit, 10^−3^ m m^−2^), and 0.00–0.73 (0.12), respectively (Table 2). Scatter plots of these three variables for studied ponds classified by predominant land use in areas surrounding ponds show that ponds surrounded predominantly by paddy fields exhibit moderate road density, but higher visibility than in areas surrounded by urban or woodland land use (Fig. 2A). In urban areas, despite higher road density, visual exposure of ponds was lower than in paddy field areas, probably because sight lines tended to be obscured by a greater number of building blocks surrounding ponds.

**Table 2:** Summary of continuous environmental characteristics for 64 agricultural ponds in Hyogo Prefecture, Japan.

Characteristics	Min.	25th %tile	Median	75th %tile	Max.
Visibility (number of viewshed points)	0	8,996	26,239	89,053	472,752
Road density (10^−3^ m m^−2^)	0.14	7.66	12.21	16.48	25.18
Urban ratio	0.00	0.03	0.12	0.30	0.73
Pond surface area (m^2^)	685	4,321	6,897	13,220	111,626
Chlorophyll *a* (µg L^−1^)	1.9	7.4	26.2	73.6	438.8
River density (10^−3^ m m^−2^)					
5 m buffer	0.00	0.00	0.00	0.00	5.93
10 m buffer	0.00	0.00	0.00	0.00	4.78
25 m buffer	0.00	0.00	0.00	0.00	9.67
50 m buffer	0.00	0.00	0.00	0.00	7.12
100 m buffer	0.00	0.00	0.00	0.00	4.71
250 m buffer	0.00	0.00	0.00	1.13	5.69
500 m buffer	0.00	0.00	0.58	1.20	3.80
1000 m buffer	0.00	0.58	0.83	1.16	1.75

Chl.*a* concentration varied greatly among ponds: 1.9–438.8 µg L^−1^ (median 26.2 µg L^−1^) (Table 2). Over 70% of all ponds exhibited eutrophic (chl.*a*: 8–25 µg L^−1^) or hypertrophic (≥25 µg L^−1^) conditions according to the trophic category of lakes [55]; 40% of all ponds had algae blooms. Water from dam reservoirs flowed into about half of the studied ponds either directly (19 ponds) or indirectly (14 ponds). The ponds from which water was drained periodically (27 ponds) were fewer than undrained ponds (37 ponds).

### Classification trees for invasive fish

**Figure 3:**
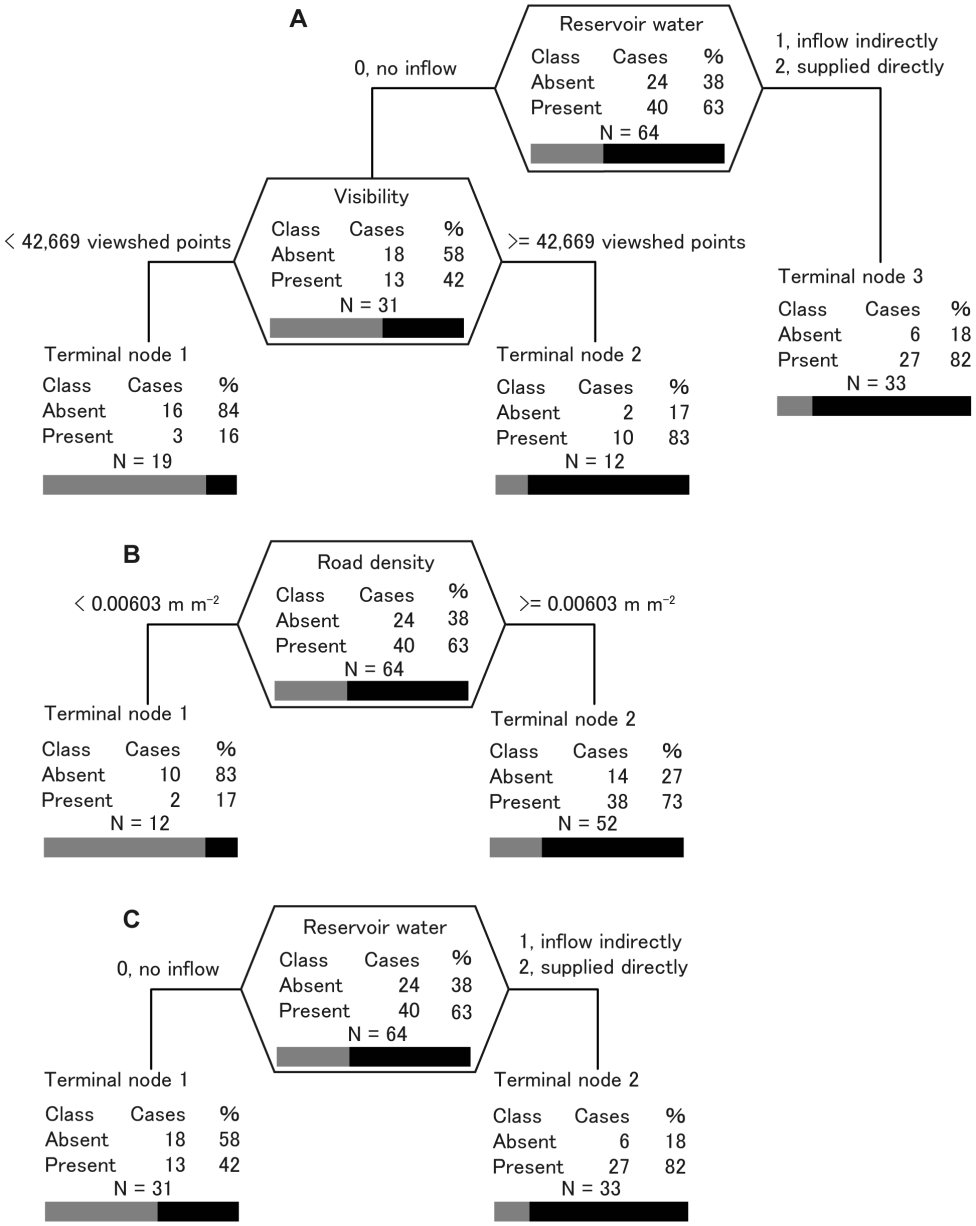
Results of classification tree analysis of the probability of Bluegill (*Lepomis macrochirus*) presence. The optimal tree is shown, which includes (A) visibility, (B) road density, and (C) urban ratio as a surrogate indicator for human-mediated introduction. %, percentage of cases for each class; bars represent the percentage of absent [gray] and present [black]. Although the urban ratio was included among environmental factors, the classification tree procedure omitted the variable from the optimal tree because of its lack of significance (C). The splitting variable name and split criterion are given for each node. The vertical depth of each node is proportional to its improvement value.

Based on the optimal tree that incorporated visibility from roads, connectivity with upstream dam reservoirs explained the greatest share of variance in the Bluegill distributions (Fig. 3A). For 82% (27 ponds) of all ponds, reservoir water, supplied directly or indirectly, supported Bluegill. Among ponds without a reservoir water connection (31 ponds), Bluegill tended to be present in ponds with higher visibility (viewshed points ≥ 42,669). The tree incorporating visibility showed the lowest misclassification value (17%) with 0.93 for sensitivity and 0.67 for specificity (Table 3), which indicated that visibility is more effective for predicting the Bluegill distribution than traditional indicators such as road density and urban ratio are. An optimal tree using road density instead of visibility revealed that ponds with higher road density (≥0.00603 m m^−2^) were more likely to have Bluegill present (Fig. 3B). This tree had the highest value of sensitivity (0.95), but the lowest value of specificity (0.42), which caused higher misclassification (25%) than the visibility tree did (Table 3). The result suggests overestimation of the presence for Bluegill by the classification tree using road density. However, an optimal tree using the urban ratio had only connectivity with upstream reservoirs as explanatory variables; the urban ratio did not explain the variance in the Bluegill distribution (Fig. 3C). This tree showed the highest misclassification value (30%) among the three models for Bluegill (Table 3).

**Table 3:** Overall misclassification, sensitivity, and specificity of optimal classification trees for the probability of Bluegill (*Lepomis macrochirus*) and Largemouth bass (*Micropterus salmoides*) presence in 64 agricultural ponds in Hyogo Prefecture using visibility, road density, and urban ratio as surrogate indicators for human-mediated introduction.

Invasive alien fish species	Indicators	Overall misclassification (%)	Sensitivity	Specificity
Bluegill (*Lepomis macrochirus*)	Visibility	17	0.93	0.67
	Road density	25	0.95	0.42
	Urban ratio	30	0.68	0.75
Largemouth bass (*Micropterus salmoides*)	Visibility	39	0.92	0.54
	Road density	39	0.92	0.54
	Urban ratio	39	0.92	0.54

**Figure 4:**
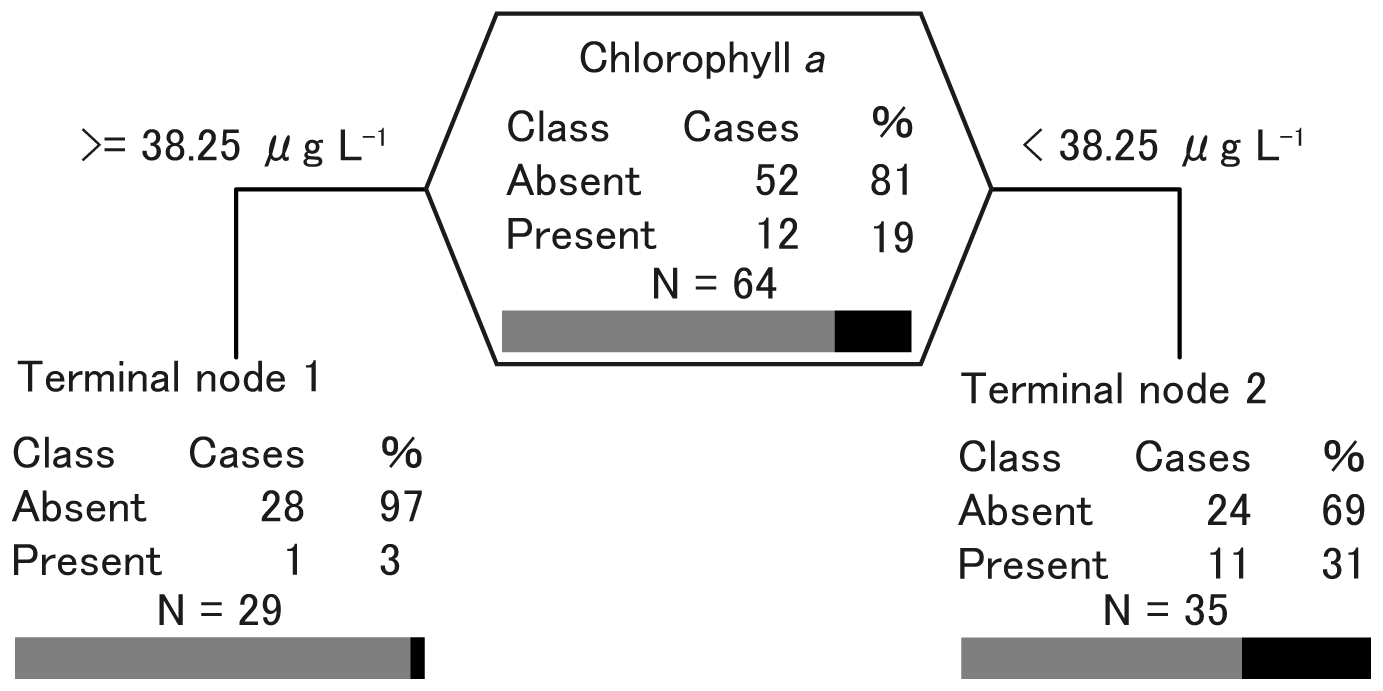
Results of classification tree analysis of the probability of Largemouth bass (*Micropterus salmoides*) presence. %, percentage of cases for each class; bars represent the percentage of absent [gray] and present [black]. Only chlorophyll *a* was used for all optimal trees irrespective of surrogate indicators for human-mediated introduction including visibility, road density, and urban ratio. The split criterion is given for each node.

For Largemouth bass, only chl.*a* presented in all optimal trees, irrespective of surrogate indicators for human-mediated introduction (visibility, road density, and urban ratio) (Fig. 4). Largemouth bass were absent from almost all ponds in which chl.*a* was higher than 38.25 µg L^−1^, corresponding to the hypertrophic category [55]. No difference of predictive power was found among indicators for Largemouth bass (Table 3). The distribution of Largemouth bass is explainable by an optimal tree model with higher misclassification (39%) than that for Bluegill.

## Discussion

### Factors affecting invasive fish species distribution

The distribution (presence and absence patterns) of Bluegill, an alien game species in agricultural ponds, was explainable by the surrogate indicators for propagule pressure including the visibility of ponds from roads and the road density surrounding ponds. The result suggests that the probability of human-mediated introduction of the species becomes higher at ponds with higher visibility or denser roads, which concurs with previous findings indicating that the spatial distribution patterns of alien game species are likely to be affected by human-mediated introduction [5], [14].

Previous studies used various indicators related to human populations (e.g. population density [6]), urban land use (e.g. proportional area of urban and industrial land [11]), and roadways (e.g. distance from roads and road use types [9], [10]) as surrogates of human-mediated introduction to predict the distribution and richness of invasive species. Our study demonstrates the effectiveness of road density in predicting the occurrence of Bluegill, although road density exhibited lower predictive power than pond visibility from roadways. An optimal tree incorporating visibility showed the lowest misclassification value, probably because visibility from roads has best represented behavioral characteristics of human related to the stocking of invasive fish species. In urban areas, although ponds are surrounded by denser road networks, visibility is apparently reduced by the many blocks of buildings that obscure the ponds. Consequently, the classification tree using only road density showed overestimation of the presence for Bluegill.

In contrast to visibility and road density, the urban ratio was not adopted for any classification tree as a predictor in our analyses. Sport anglers are generally known to come not from the immediate proximity of the ponds but from more populated urban areas such as southern coastal regions and metropolitan areas on the eastern edge of the study area. At a local scale with the study area of approximately 30 × 30 km, all ponds within the study area are assumed to be included in the home range for people who live in these populated areas. Therefore urban ratios are not an indicator of the potential accessibility to the ponds. The ratios can not explain the distribution of the invasive fish. Nevertheless, it is clear that urban land use as well as human populations can be strong predictors of species invasions at a larger extent (e.g. country or province scales) as described in many reports of the literature [5], [6], [11]. These results suggest that suitable predictors differ on a spatial scale. The indicators which directly characterize human behaviors related to propagule supply are more effective for local assessments.

For predicting the present distribution of invasive species, the cumulative effect of human-mediated introduction from the initial introduction to the present should be considered. According to the 1/25,000 topographic map (provided by the GSI) and Land utilization segmented mesh data (provided by the MLIT), the landforms and land use surrounding the study ponds did not change considerably after the late 1980s when Bluegill were first introduced into this area. This observation suggests that the visibility of ponds from roads has not changed considerably in the past few decades. Consequently, the present visibility can be regarded as an appropriate indicator of human-mediated introduction from the initial introduction to the present.

Irrespective of visibility, Bluegill tended to be present in ponds to which reservoir water was supplied directly or indirectly. Given that invasive fish species including Bluegill are observed in dam reservoirs in the study area, Bluegill seem to spread spontaneously from upstream established reservoirs via irrigation canals. This finding supports the general notion that dams facilitate biological invasion into freshwater bodies by creating additional habitats with hydrological alterations, fish stocking, and secondary spread to surrounding water bodies [56].

Consequently, variance in Bluegill distributions was explained well solely by propagule supplies from ponds' peripheral areas, including visibility and connectivity with upstream reservoirs. Their distributions were not constrained by any site characteristic, probably because they have high adaptability to environments (see Methods) and readily establish in any pond into which they are introduced. Drainage of pond water did not affect their distributions, probably because they are introduced again even if they are eradicated by pond drying [57], which suggests that pond draining is probably ineffective as a control or eradication technique.

In contrast, the Largemouth bass distribution was explained by a site characteristic: they were absent from hypertrophic ponds. Bonvechio and Bonvechio [58] reported negative correlation between the population of Largemouth bass and chl.*a* concentration in their native range, which is assumed to result from the lack of dissolved oxygen [59] with the decomposition of plenty of organic matter and decrease in foraging success with higher turbidity [60]. Largemouth bass are likely to be constrained by site characteristics because of their lower adaptability to environments than that of Bluegill [61].

Generally, because invasive species have a larger niche space and greater environmental tolerance, they spread naturally from introduced sources to non-invaded sites that possess suitable environmental conditions if propagule supplies are sufficient to sustain their populations [62]. Consequently, their distributions in invasion areas tend to be limited by the environmental conditions of sites. The tendency is regarded as strengthening as time passes after their initial introduction, with decreasing relative importance of propagule pressure on their distributions [63]. However, in the present study, propagule pressure dominantly explained the Bluegill distribution despite nearly 20 years since their initial introduction into the study area. This result is attributed to poor connectivity among ponds for their natural dispersal. Dispersion corridors are limited to waterways such as rivers, streams, and canals. Invasive fish might only rarely have sufficiently active (fish-vectored) transport to upstream ponds through vertical differences of a few meters between ponds and downstream drainage canals [40]. These results suggest that, for invasive species with limited natural dispersibility, the quantification of propagule pressure might be effective for predicting their distributions even if a long time passes after their initial introduction.

### Efficient management of invasive species and future studies

The efficient management of invasive species demands knowledge of which factor, propagule pressure or site characteristics, is most important for their distribution [64]. Regarding our studied fish species, the Bluegill distribution depended most strongly on the indicators for propagule pressure including visibility from roads and connectivity with upstream reservoirs. Consequently, inhibiting propagule supplies into ponds is probably efficient for preventing any further invasion. These indicators used in the classification tree can be readily assessed for all agricultural ponds in the study area, which enables prediction of which areas will be susceptible to introduction of the species. Recently, international recognition has been given to the importance of local biodiversity strategies as a means of complementing and supporting national biodiversity actions and of contributing to the implementation of a strategic plan for biodiversity [2]. Our results will be useful for local planning in terms of controlling biological invasions.

The visibility of a site and its contribution to site accessibility and visitation are applicable to various alien animal and plant species that spread with frequent instances of human-mediated introduction. For example, angling is the major driver of fish species introduction worldwide [5], [65]. Incidental introduction of aquatic organisms (e.g. sessile invertebrates and macrophytes) via contaminated boating gear, nets, and other equipment is common in many inland water bodies [6], [66]. Visibility might be useful to detect areas of high invasions risk with higher accessibility by vehicles and recreational watercraft. Additionally, increasing accessibility facilitates species invasion into mountainous areas by residents and tourists [10], [12]. It is possible for visibility to become an efficient predictor for species invasions not only in inland water areas but also in terrestrial ecosystems.

Traditionally, the information associated with potential distribution and relative abundance of invasive species predicted by ecological niche modeling has been widely used for the planning of invasion control efforts [62], [64]. However, the availability is assumed to be lower for species that depend strongly on human-mediated dispersion. In this case, predicting their distributions based on an analysis of human behaviors must be more important, demanding indicators for quantifying the behavioral characteristics affected by their psychographic and sociological conditions.

Biological invasion studies particularly addressing behavioral characteristics of human-mediated introduction are very few. Buchan and Padilla [66] examined the relations between the recreational boater movement and invasive zebra mussel patterns in Wisconsin lakes. Niggemann et al. [67] studied the effect of human behavior between settlements on invasive plant dispersion in Germany, based on questionnaire surveys and statistical data. However, such information is generally lacking, leading to a lack of versatility [6]. Recently, various social information (e.g., population and industrial structure) as well as digital map information (e.g., DSMs, Digital Elevation Models [DEMs], and road networks) has become increasingly available as spatial information with large scale and higher resolution. Furthermore, technological advances in data processing using GIS engender increasing development and application of spatial analysis [68]. With these advancements, it has become possible to quantify human behaviors more directly, objectively, and broadly with spatial indicators including visibility, connectivity and travel costs through transportation networks [69], [70]. Using these indicators, control measures for biological invasions are expected to become more effective.
